# Data on European non-residential buildings

**DOI:** 10.1016/j.dib.2017.08.043

**Published:** 2017-09-01

**Authors:** Delia D'Agostino, Barbara Cuniberti, Paolo Bertoldi

**Affiliations:** European Commission, Joint Research Centre (JRC), Directorate C -- Energy, Transport and Climate, Energy efficiency and Renewables Unit, Via E. Fermi 2749, I-21027 Ispra, VA, Italy

## Abstract

This data article relates to the research paper Energy consumption and efficiency technology measures in European non-residential buildings (D'Agostino et al., 2017) [1]. The reported data have been collected in the framework of the Green Building Programme that ran from 2006 to 2014. The project has encouraged the adoption of efficiency measures to boost energy savings in European non-residential buildings. Data focus on the one-thousand buildings that joined the Programme allowing to save around 985 GWh/year. The main requirement to join the Programme was the reduction of at least 25% primary energy consumption in a new or retrofitted building. Energy consumption before and after the renovation are provided for retrofitted buildings while, in new constructions, a building had to be designed using at least 25% less energy than requested by the country's building codes. The following data are linked within this article: energy consumption, absolute and relative savings related to primary energy, saving percentages, implemented efficiency measures and renewables. Further information is given about each building in relation to geometry, envelope, materials, lighting and systems.

**Specifications Table**

**Table:** 

Subject area	*Engineering*
More specific subject area	*Buildings*
Type of data	*Table*
How data was acquired	*Data collection*
Data format	*.xls*
Experimental factors	*no data pretreatment*
Experimental features	*Data on non-residential European buildings collected between 2006 and 2014 in the framework of the Green Building Programme*
Data source location	*European Member States*
Data accessibility	*Data are provided in supplementary materials directly with this article*

**Value of the data**

• The data give quantitative information on European non-residential buildings.
• The data provide information on energy savings and implemented solutions in new and existing buildings.
• The data can be used for comparison with other building data or further analysis.
• The data support energy efficiency and energy policies implementation at European level [2].
• The data give insight on technological measures adopted across Europe in buildings.

## 1 Data

An excel spreadsheet reports the collected data related to the buildings that joined the Green Building Programme between 2006 and 2014. About one-thousand buildings have been welcomed by the project during its operation collecting both new and refurbished buildings.

Data refer to both new and existing non-residential buildings. Data include the main building characteristics starting from its general description. Geometrical and geospatial building features are also included (e.g. partner, area, year of construction, building type and category). The data collected comprise energy consumption, energy savings and implemented measures. These are reported in absolute (kWh/y) and relative terms (kWh/m^2^/y) as well as in terms of percentage of savings (%). In the linked database, more details are given on envelope, systems, technologies, lighting, and renewables.

## 2 Experimental design, materials and methods

Non-residential buildings account for 25% of the European building stock [3] and represent a heterogeneous sector compared with the residential [4]. The data reported in this article have been collected in the framework of the Green Building Programme during nine years: from 2006 to 2014. The project encouraged the adoption of measures to boost energy savings in European non-residential buildings on a voluntary basis. The data collection was managed by the Joint Research Centre (JRC) of the European Commission and it has been operational in European Member States as well as European Economic Area countries [5].

The Programme welcomed both new and refurbished buildings. The main requirement to join the project was the reduction of at least 25% primary energy in a new or retrofitted building. Refurbishing activities could involve the whole building or part of it. In case of new buildings, the achieved primary consumption had to be 25% below the building code in force in the building's country. In the case of refurbishment, the energy consumption of the proposed building had to show at least 25% of energy improvement before and after the intervention.

A specific procedure had to be followed to become a partner of the Green Building Programme. This started from an energy audit performed by a building owner followed by an action plan defining the scope and nature of a project. A report had to detail the improvements made to the performance of a building as well as information on achieved savings and a description of the implemented measures. Before being accepted within the Programme, submitted applications have been checked for consistency and quality.

The collected data have been used for statistical analysis, periodical evaluations, catalogues, and reports [6], [7]. Table 1 of [1] reports the main categories of the GPB in relation to prevalent building use.

The data of this article relate to the 533 partners that took part in the project during its operation. As reported in Fig. 3 of [1], a building proposal has to be submitted with a specific form that had to detail the building project. Each partner filled in an application giving information and data about the building, such as achieved savings and energy efficiency measures through which these have been achieved.

For each building, the following elements are detailed in the linked spreadsheet: absolute and relative savings related to primary energy, saving percentages, implemented measures and renewables. In order to be harmonized among European Member States, the implemented energy efficiency measures have been divided into: heating, cooling, ventilation, lighting, RES, control system, and envelope (walls and windows).

Other information available in the linked database is: name of the partner, partner website, welcome year, country, building use, and category. The project type (refurbished or new) is also given together with the year of construction and the area.

Description on geometry, materials, systems, and on-site renewable generation is also given. In more details, type of envelope, heating, HVAC, control systems, lighting are specified.

Energy consumptions are compared to the pre-refurbishment state in existing buildings. Previous consumption is specified in the datasheet. Values in kWh/y for primary energy consumption are given following the disaggregation according to standard EN 15603 [8]. These values related to the pre-refurbishment state can be compared to the values related to the final status. In this way absolute, relative and percentage savings can be calculated.
